# Black box no more: a scoping review of AI governance frameworks to guide procurement and adoption of AI in medical imaging and radiotherapy in the UK

**DOI:** 10.1259/bjr.20221157

**Published:** 2023-10-03

**Authors:** Nikolaos Stogiannos, Rizwan Malik, Amrita Kumar, Anna Barnes, Michael Pogose, Hugh Harvey, Mark F McEntee, Christina Malamateniou

**Affiliations:** 1 Discipline of Medical Imaging and Radiation Therapy, University College Cork, Cork, Ireland; 2 Division of Midwifery & Radiography, City, University of London, London, United Kingdom; 3 Medical Imaging Department, Corfu General Hospital, Corfu, Greece; 4 Bolton NHS Foundation Trust, Farnworth, United Kingdom; 5 Frimley Health NHS Foundation Trust, Frimley, United Kingdom; 6 King’s Technology Evaluation Centre (KiTEC), School of Biomedical Engineering & Imaging Science, King’s College London, London, United Kingdom; 7 Hardian Health, Bolton, United Kingdom; 8 School of Health Sciences (HESAV), University of Applied Sciences and Arts Western Switzerland (HES-SO), Lausanne, Switzerland

## Abstract

Technological advancements in computer science have started to bring artificial intelligence (AI) from the bench closer to the bedside. While there is still lots to do and improve, AI models in medical imaging and radiotherapy are rapidly being developed and increasingly deployed in clinical practice. At the same time, AI governance frameworks are still under development. Clinical practitioners involved with procuring, deploying, and adopting AI tools in the UK should be well-informed about these AI governance frameworks. This scoping review aimed to map out available literature on AI governance in the UK, focusing on medical imaging and radiotherapy. Searches were performed on Google Scholar, Pubmed, and the Cochrane Library, between June and July 2022. Of 4225 initially identified sources, 35 were finally included in this review. A comprehensive conceptual AI governance framework was proposed, guided by the need for rigorous AI validation and evaluation procedures, the accreditation rules and standards, and the fundamental ethical principles of AI. Fairness, transparency, trustworthiness, and explainability should be drivers of all AI models deployed in clinical practice. Appropriate staff education is also mandatory to ensure AI’s safe and responsible use. Multidisciplinary teams under robust leadership will facilitate AI adoption, and it is crucial to involve patients, the public, and practitioners in decision-making. Collaborative research should be encouraged to enhance and promote innovation, while caution should be paid to the ongoing auditing of AI tools to ensure safety and clinical effectiveness.

## Introduction

Artificial intelligence (AI) is increasingly employed in healthcare.^1^ Recent technology and neuroscience breakthroughs brought AI from the lab to the clinic.^2^ Medical imaging was one of the first disciplines to adopt AI technologies,^3^ with AI-enabled applications being used for pathology detection and staging, image reconstruction, segmentation, image optimisation, automation and optimisation of workflows, automation of imaging protocols, feature extraction etc., to name just a few.^4–8^

While AI-driven applications for use in clinical practice are increasing, there is, in parallel exponential interest and discussion on the need for rigorous AI governance frameworks.^9^ AI governance may entail processes related to the ethical use and deployment of AI tools, regulation and accreditation of AI models, liability, accountability, data protection processes, and education, among others.

In medical imaging, the implementation of robust AI governance frameworks is required for the safe adoption of AI in clinical practice.

Although AI is a ubiquitous term, AI governance remains loosely defined and largely underdeveloped, with no consensus on what AI governance might entail.^10^ Recent research has shown important variation among organisations and countries regarding governance. A lack of standardisation could impede AI adoption, create market disparities, and compromise safety.^11^ Processes like procurement, validation and evaluation, monitoring and decommissioning, as part of the AI product lifecycle, are all impacted by the lack of standardised AI governance frameworks. Hence, there is an urgent need to propose a robust, unified governance framework to enhance the trustworthiness and transparency of AI systems and mitigate any potential risks associated with the implementation of AI-enabled solutions.^12,13^

In medical imaging departments, healthcare professionals, including radiographers, radiologists, and medical physicists, are often responsible for procuring medical imaging equipment. Multidisciplinary teams also ensure the safe running of the equipment by implementing robust quality assurance programmes and escalating concerns, as required. Given the ongoing increase of AI tools in clinical imaging, radiographers, radiologists, medical physicists and other relevant professionals are expected to acquire substantial knowledge related to AI in medical imaging and radiotherapy, to be in a position to facilitate clinical adoption.^14–17^ Different regulatory and professional bodies in healthcare and medical imaging are pushing for AI competencies becoming central to healthcare practitioners’ training.^18–22^

Scoping reviews are ideal for exploring emerging literature on fast-developing topics and identifying knowledge gaps.^23^ This scoping review, part of a more comprehensive research project exploring the notion of AI governance in medical imaging and radiotherapy, aims to map out all currently available literature (peer-reviewed and grey literature) in these fields and propose a comprehensive AI governance framework.

The research question that guides this scoping review is “What might be relevant to an AI governance framework in medical imaging and radiotherapy in the UK?”. This question was generated in line with the Population, Concept, Context framework for scoping reviews.^24^

## Methods

### Review protocol

This article is structured in line with the Preferred Reporting Items for Systematic reviews and Meta-Analyses extension for Scoping Reviews (PRISMA-ScR) checklist.^25^

An explicit review protocol was followed for inclusion and exclusion criteria, data extraction methods and objectives.^24^ Ethical approval was obtained from City, University of London School of Health and Psychological Sciences Research Ethics Committee (ref: ETH2122-1015).

### Eligibility criteria

Table 1 below demonstrates the eligibility criteria applied to this scoping review.

**Table 1.** Eligibility criteria

Studies published within the last 5 years (2017–2022).
Studies published only in the English language.
Full-text articles only.
Both peer-reviewed studies and grey literature (white papers, guidelines, guidance, standards, and regulations) will be included.
All study designs were eligible for this review.

### Information sources

The following databases were searched: a) Google Scholar, b) PubMed, and c) The Cochrane Library. Searches were initiated on 30 May 2022, and the last search was performed on 17 June 2022. A new search was also performed on 15 October 2022, to ensure we captured any latest papers.

### Search

A consistent methodology was applied for all database searches to enhance the reproducibility of this scoping review.^26^ All searches were performed using explicit, pre-defined keywords related to the topic under exploration. Appropriate search strings were developed using Boolean operators ‘’AND’’ and ‘’OR’’ to narrow down the results. A completely worked example of the search strategy for one database is provided as Supplementary Material. The pearl growing search technique was also applied to identify relevant sources of evidence in the reference lists of already obtained studies.

Supplementary Material 1.

### Selection of sources of evidence

A researcher (NS) initially screened the articles at the level of titles and abstracts to identify relevant/non-relevant results. Non-relevant articles were excluded at this stage, based on the content provided in titles/abstracts, and only relevant articles were assessed for eligibility based on inclusion and exclusion criteria (full-text evaluation). All results were saved on the Zotero reference manager, v. 6.0.13 (Corporation for Digital Scholarship, Virginia), for further evaluation, and duplicates were automatically removed. All relevant studies were read thoroughly and evaluated against the eligibility criteria. A senior researcher (CM) then advised on the final study selection, and that list was reviewed by the research team and finalised based on consensus reading amongst the researchers.

### Data charting process

To extract all meaningful data from the eligible studies, a data-charting form was used to allow a visual map of the included studies and enable correlations and convergence of ideas and topics.^27^ This charting table (Table 2) allowed for a standardised method of data charting, and any disagreements were resolved until a consensus was reached.

**Table 2.** Data extraction form

Title	Author(s)	Year	Country	Aim/purpose	Population/ sample	Methods	Intervention type	Duration of intervention	Key findings related to the scoping review question
A governance model for the application of AI in health Care.	Reddy et al.	2020	Australia	To propose an AI governance model.	n/a	Narrative review	n/a	n/a	Key ethical challenges (privacy, patient/clinician trust). Algorithm biases. Regulatory concerns. Fairness, transparency, trustworthiness, accountability. Liability issues. XAI.
A guide to good practice for digital and data-driven health technologies.	Department of Health & Social Care	2021	UK	To support innovators in understanding what the NHS is looking for when it buys digital and data-driven technology for use in health and care.	n/a	Grey literature	n/a	n/a	Ethics. Understanding users’ needs. Ensure usability and accessibility. Validation testing. Ensure clinical safety. Data protection. Fairness, transparency. Cybersecurity. Algorithmic biases. Regulation (UK). Interoperability. Data standards.
Evidence standards framework (ESF) for digital health technologies.	National Institute for Health and Care Excellence.	2022	UK	For companies that develop or distribute, and for evaluators and decision makers in the health and care system.	n/a	Grey literature	n/a	n/a	Classification of DHTs by intended purpose. Design factors (safety, technical standards, reliability). Intended purpose and target population. Expected cost, health, and resource impacts. Ensure DHTs effectiveness. Evaluate changes over time. Cost-effectiveness analysis. Ensure transparency during deployment. Ensure appropriate scalability. Communication and education for end users.
Ethics Guidelines for trustworthy AI.	European Commission	2019	Belgium	To promote trustworthy AI and set out a framework to achieve this.	n/a	Grey literature	n/a	n/a	Ethics (human autonomy, fairness, no harm, explicability, attention to vulnerable groups). 7 key requirements for trustworthy AI (human agency and oversight, technical robustness and safety, privacy and data governance, transparency, diversity, non-discrimination and fairness, environmental and societal well-being, and accountability. Effectively communicate information to stakeholders. Train new experts on AI. Foster research and innovation. Adopt a trustworthy AI assessment list when developing, deploying, or using AI systems, and adapt it to the specific use case in which the system is being applied.
Artificial intelligence in hospitals: providing a status quo of ethical considerations in academia to guide future research.	Mirbadaie et al.	2021	Germany	To identify the status quo of interdisciplinary research in academia on ethical considerations and dimensions of AI in hospitals.	15 articles published in medical journals.	Systematic discourse	n/a	n/a	Transparency Trustworthiness Fairness Liability Accountability Explainability Education of workforce Patient safety Privacy Informed consent Security Interoperability Beneficence, justice, autonomy, non-maleficence.
Artificial Intelligence and Healthcare Regulatory and Legal Concerns.	Ganapathy K.	2021	India	To discuss liability issues when AI is deployed in healthcare.	n/a	Narrative Review	n/a	n/a	XAI. Data protection. Trustworthy AI. Informed consent. Regulation (IMDRF and FDA). Liability concerns.
Artificial Intelligence and Liability in Medicine: Balancing Safety and Innovation.	Maliha et al.	2021	USA	To discuss liability issues.	n/a	Narrative Review	n/a	n/a	Regulation (FDA). Liability issues (negligence, medical malpractice, product liability). Legislation (USA).
Artificial intelligence and medical imaging 2018: French Radiology Community white Paper.	SFR-IA Group, CERF	2018	France	To issue a position paper on AI.	n/a	Narrative Review	n/a	n/a	Regulation (GDPR, French legislation). Data protection, pseudonymization. Research (France). Education (radiologists only). Algorithm biases. Ethics.
Artificial intelligence as a medical device in radiology: ethical and regulatory issues in Europe and the United States.	Pesapane et al.	2018	Binational	To analyse the legal framework regulating medical devices and data protection in Europe and in the United States.	n/a	Narrative Review	n/a	n/a	Regulation (EU and USA). Data protection (EU and USA). Accountability.
Artificial Intelligence in Cardiovascular Imaging: “Unexplainable” Legal and Ethical Challenges?	Lang et al.	2022	Canada	To discuss legal and ethical issues arising from unexplainable AI models.	n/a	Narrative Review	n/a	n/a	XAI Regulation (Canada and USA). Liability. Data protection (EU).
Artificial intelligence in healthcare: a critical analysis of the legal and ethical implications.	Schonberger D.	2019	UK	To discuss ethical and legal challenges of AI in healthcare.	n/a	Narrative review	n/a	n/a	Regulation (UK, EU, USA). Fairness. Non-discrimination laws (UK and EU). Respect for autonomy laws (UK and EU). Informed consent (UK). GDPR Accountability laws (EU). Liability (UK). Transparency.
Artificial Intelligence in Radiology— Ethical Considerations.	Brady & Neri	2020	Multinational	To explain some of the ethical challenges, and some of the measures we may take to protect against misuse of AI.	n/a	Narrative review	n/a	n/a	Data ownership and privacy. GDPR. Informed consent. Data anonymization. Data biases Transparency Interpretability Explainability Resource inequality. Liability (EU) Explicability
Canadian Association of Radiologists White Paper on Artificial Intelligence in Radiology.	Tang et al.	2018	Binational	To inform CAR members and policymakers on key terminology, educational needs of members, research and development, partnerships, potential clinical applications, implementation, structure and governance, role of radiologists, and potential impact of AI on radiology in Canada.	n/a	Narrative Review	n/a	n/a	Education Data anonymization. Data sharing Data biases Interoperability Multidisciplinary teams engaged.
Checklist for Artificial Intelligence in Medical Imaging (CLAIM): A Guide for Authors and Reviewers.	Mongan et al.	2020	USA	To propose CLAIM, the Checklist for AI in Medical Imaging.	n/a	Narrative Review	n/a	n/a	-Guidelines for reporting AI studies.
DECIDE-AI: new reporting guidelines to bridge the development to implementation gap in clinical artificial intelligence.	The DECIDE-AI Steering Group.	2021	Multinational	To propose guidelines to report key information items between the *in silico* algorithm development/validation and large-scale clinical trials evaluating AI interventions.	n/a	Narrative review	n/a	n/a	-Reporting guidelines.
Do no harm: a roadmap for responsible machine learning for health care.	Wiens et al.	2019	Binational	To provide a comprehensive overview of the barriers to deployment and translational impact.	n/a	Narrative review	n/a	n/a	Ethics Algorithm biases Data biases Model evaluation Validation Reporting guidelines. Regulation (USA)
Emerging Consensus on ‘Ethical AI’: Human Rights Critique of Stakeholder Guidelines.	Fukuda-Parr & Gibbons	2021	USA	To review 15 guidelines preselected to be strongest on human rights, and on global health.	15 guidelines	Narrative review	n/a	n/a	Respect to privacy and freedom of expression. Human rights laws (international). Universality Accountability Equality Participation Informed consent Regulation (EU, USA, Canada).
Ethical and legal challenges of informed consent applying artificial intelligence in medical diagnostic consultations.	Astromske et al.	2021	Lithuania	To discuss the process of informed consent when using AI.	n/a	Narrative review	n/a	n/a	Informed consent Trustworthiness Informed consent legislation (EU and USA). GDPR
Ethical considerations for artificial intelligence: an overview of the current radiology landscape.	D'Antonoli TA	2020	Switzerland	To discuss ethical issues around AI.	n/a	Narrative review	n/a	n/a	Algorithm biases Transparency Accountability Multidisciplinary stakeholders involved. Regulation (EU, USA, Canada). Data privacy Education Liability Explicability Autonomy
Ethics of Artificial Intelligence in Radiology: Summary of the Joint European and North American Multisociety Statement.	Geis et al.	2019	Multinational	Summary of a joint statement on AI Ethics.	n/a	Narrative review	n/a	n/a	Transparency Accountability Informed consent Data privacy Data ownership Algorithm biases Cybersecurity GDPR Trustworthiness Liability
Ethics of Using and Sharing Clinical Imaging Data for Artificial Intelligence: A Proposed Framework.	Larson et al.	2020	USA	To propose an ethical framework for using and sharing clinical data for the development of AI applications.	n/a	Narrative review	n/a	n/a	Ethics Privacy of data Data protection Legislation (USA) Informed consent
Evaluation and Real-World Performance Monitoring of Artificial Intelligence Models in Clinical Practice: Try It, Buy It, Check It.	Allen et al.	2021	USA	To discuss why regulatory clearance alone may not be enough to ensure AI will be safe and effective in all radiological practices.	n/a	Narrative review	n/a	n/a	Validation Model evaluation Regulation (USA) Performance monitoring.
FUTURE-AI: Guiding Principles and Consensus Recommendations for Trustworthy Artificial Intelligence in Medical Imaging.	Lekadir et al.	2021	Multinational	To introduce a careful selection of guiding principles drawn from the accumulated experiences, consensus, and best practices from five large European projects on AI in Health Imaging.	n/a	Narrative review	n/a	n/a	Fairness Universality Traceability Usability Robustness Explainability
Identifying Ethical Considerations for Machine Learning Healthcare Applications.	Char et al.	2020	USA	To identify ethical concerns of ML healthcare applications.	n/a	Narrative review	n/a	n/a	Transparency Accountability Informed consent Justice Auditability Diverse stakeholders. Data biases
A Buyer’s Guide to AI in Health and Care.	NHSx	2020	UK	To offer practical guidance on the questions to be asking before and during any AI procurement exercise in health and care.	n/a	Grey literature	n/a	n/a	Regulation (UK) Validation Model evaluation Ethics Data protection Fairness Transparency
Privacy in the age of medical big data.	Price & Cohen	2019	Binational	To discuss patient privacy, consent, and data collection.	n/a	Narrative review	n/a	n/a	Data privacy Informed consent Data sharing
Regulatory Frameworks for Development and Evaluation of Artificial Intelligence–Based Diagnostic Imaging Algorithms: Summary and Recommendations.	Larson et al.	2021	Binational	To review the major regulatory frameworks for software as a medical device applications, identify major gaps, and propose additional strategies to improve the development and evaluation of diagnostic AI algorithms.	n/a	Narrative review	n/a	n/a	Regulation (EU, USA, International). Validation Model testing in all relevant domains. Continuous monitoring of performance. Algorithm durability. Assessment by third-party evaluators.
Reporting guidelines for clinical trials of artificial intelligence interventions: the SPIR IT-AI and CONSORT-AI guidelines.	Ibrahim et al.	2021	Binational.	To build new guidelines to report clinical trials of AI interventions.	n/a	Narrative review	n/a	n/a	-Reporting guidelines
The European artificial intelligence strategy: implications and challenges for digital health.	Cohen et al.	2020	Multinational	To present the challenges associated with the European Commission white paper on AI.	n/a	Narrative review	n/a	n/a	GDPR Regulation (EU and USA). Data privacy Algorithm update problems. Fairness Accountability
The proof of the pudding: in praise of a culture of real-world validation for medical artificial intelligence.	Cabitza & Zeitoun	2019	Binational	To propose four types of validity corresponding to different perspectives to evaluate true clinical validity.	n/a	Narrative review	n/a	n/a	Model validation Statistical validity, relational validity, pragmatic validity, ecological validity.
The roadmap to an effective AI assurance ecosystem.	Centre for Data Ethics and Innovation	2021	UK	To set out the steps needed to grow a mature AI assurance industry.	n/a	Grey literature	n/a	n/a	Trustworthiness Assessment Cybersecurity (UK). Regulation (UK) Performance testing.
To buy or not to buy—evaluating commercial AI solutions in radiology (the ECLAIR guidelines).	Omoumi et al.	2021	Multinational	To propose a practical framework that will help stakeholders evaluate commercial AI solutions in radiology and reach an informed decision.	n/a	Narrative review	n/a	n/a	Model performance. Validation Usability Interoperability Interpretability Regulation (UK, EU, USA). Financial issues
Towards a framework for evaluating the safety, acceptability and efficacy of AI systems for health: an initial synthesis.	Morley et al.	2021	UK	To set out a minimally viable framework for evaluating the safety, acceptability and efficacy of AI systems for healthcare.	n/a	Narrative review	n/a	n/a	Transparency Validation Clinical efficacy tests. Ongoing monitoring. Robustness
Understanding healthcare workers’ confidence in AI.	NHS AI Lab & Health Education England	2022	UK	To explore the factors influencing healthcare workers’ confidence in AI technologies and how these can inform the development of related education and training.	n/a	Grey literature	n/a	n/a	Regulation (UK) Evaluation Validation Trustworthiness Confidence Liability
Digital Technology Assessment Criteria (DTAC)	NHS England	2021	UK	To give patients and staff the confidence that the digital tools they use meet NHS’ clinical safety, data protection, technical security, interoperability and usability and accessibility standards.	n/a	Grey literature	n/a	n/a	Ensure clinical safety. Data protection Cybersecurity Interoperability criteria. Usability

### Data items

The team extracted essential study characteristics related to author(s), year of publication, country of origin, study population, and sample size. At the same time, any critical data relevant to the scoping review question was also extracted.

### Data analysis and synthesis of results

All included studies were coded and then grouped according to their content (*e.g.* ethics, regulation, validation etc.). Content analysis was performed to identify concepts, categories, and themes^28^ of AI governance included in the eligible papers. These themes were then compiled to inform a conceptual framework loosely based on previous clinical governance frameworks in the UK.^29–31^

## Results

### Selection of sources of evidence

**Figure 1.**
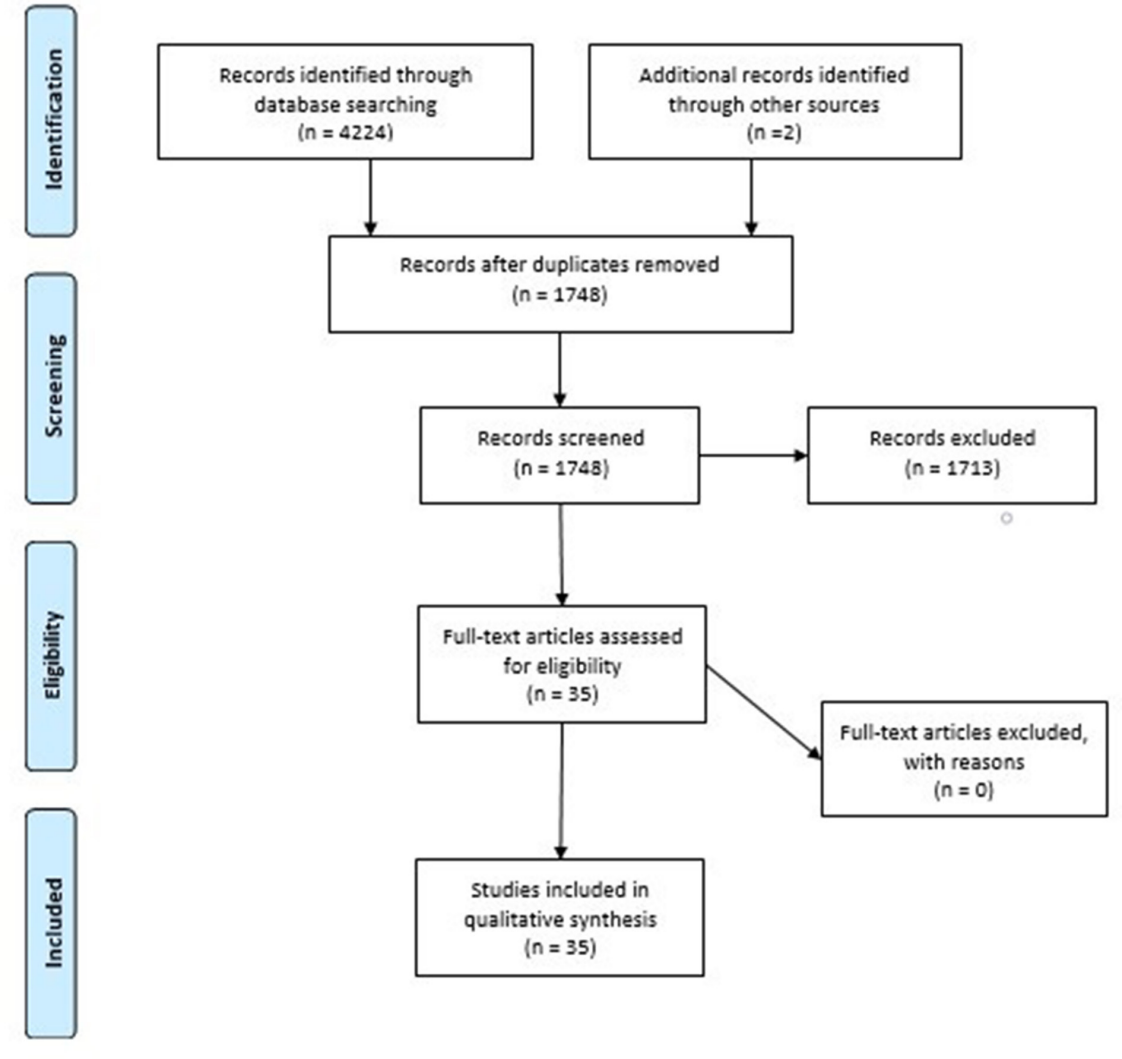
PRISMA flow diagram. PRISMA, Preferred Reporting Items for Systematic reviews and Meta-Analyses.

In total, 35 articles were included in this scoping review. The following diagram (Figure 1) demonstrates the details related to the search process, screening of articles, and final selection.

**Figure 2.**
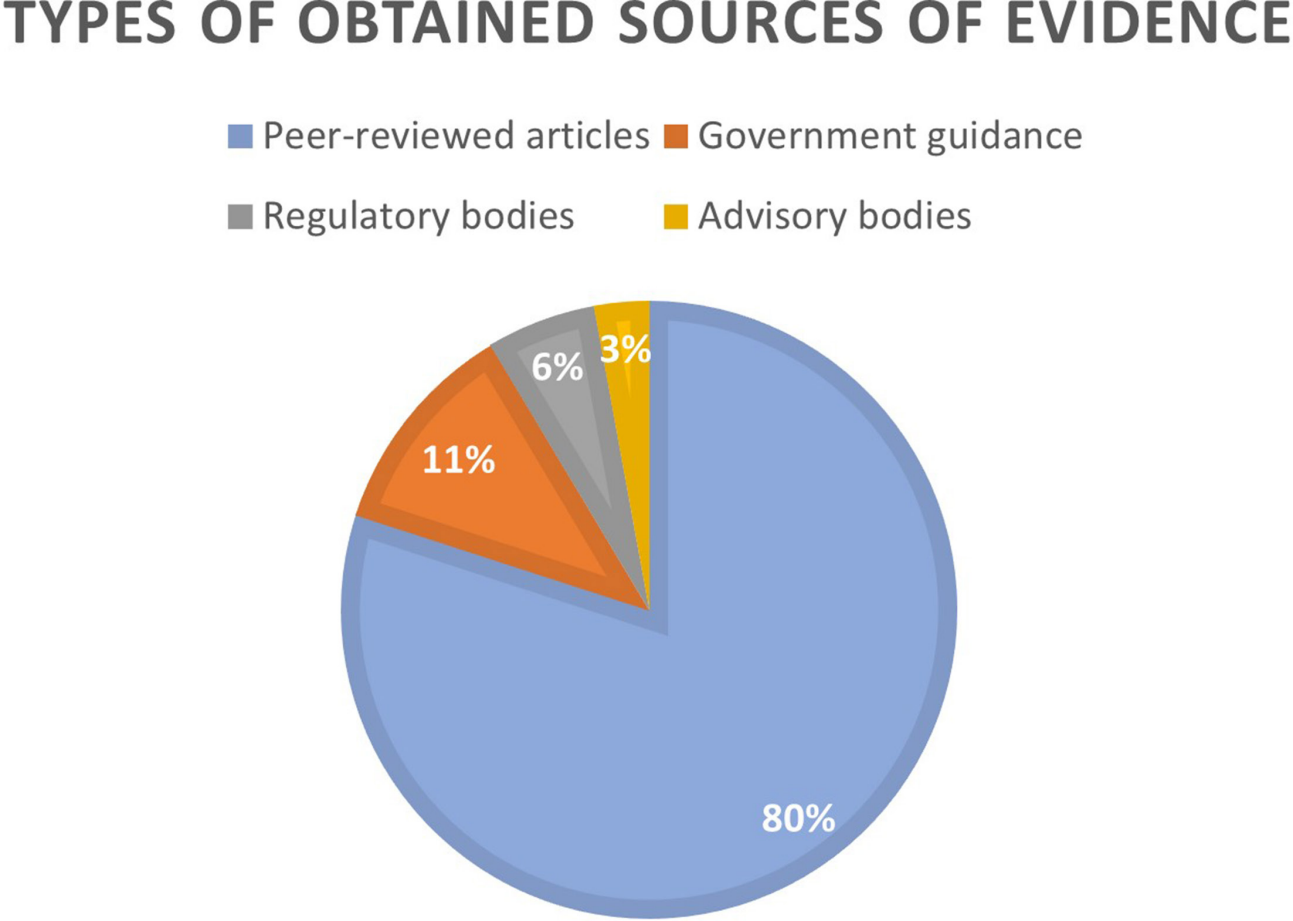
Types of the obtained sources of evidence.

Out of 35 sources of evidence included in this scoping review, 28 articles were identified as reviews, while 7 related to ‘grey literature’ (government guidance and guidelines issued by regulatory, advisory, or professional bodies). In terms of geographical distribution, the obtained studies were from the UK (*n* = 8), USA (*n* = 6), EU (*n* = 5), Canada (*n* = 1), Australia (*n* = 1), India (*n* = 1), while a further 13 of them were identified as binational/multinational. With regard to year of publication, many of them were published in 2021 (*n* = 15), followed by those in 2020 (*n* = 8), 2019 (*n* = 6), 2018 (*n* = 3) and 2022 (*n* = 3). Figure 2 provides information on the types of the obtained sources of evidence.

The main outcome of this study was to construct an AI governance framework, based on published evidence.

### Suggested AI governance framework

**Figure 3.**
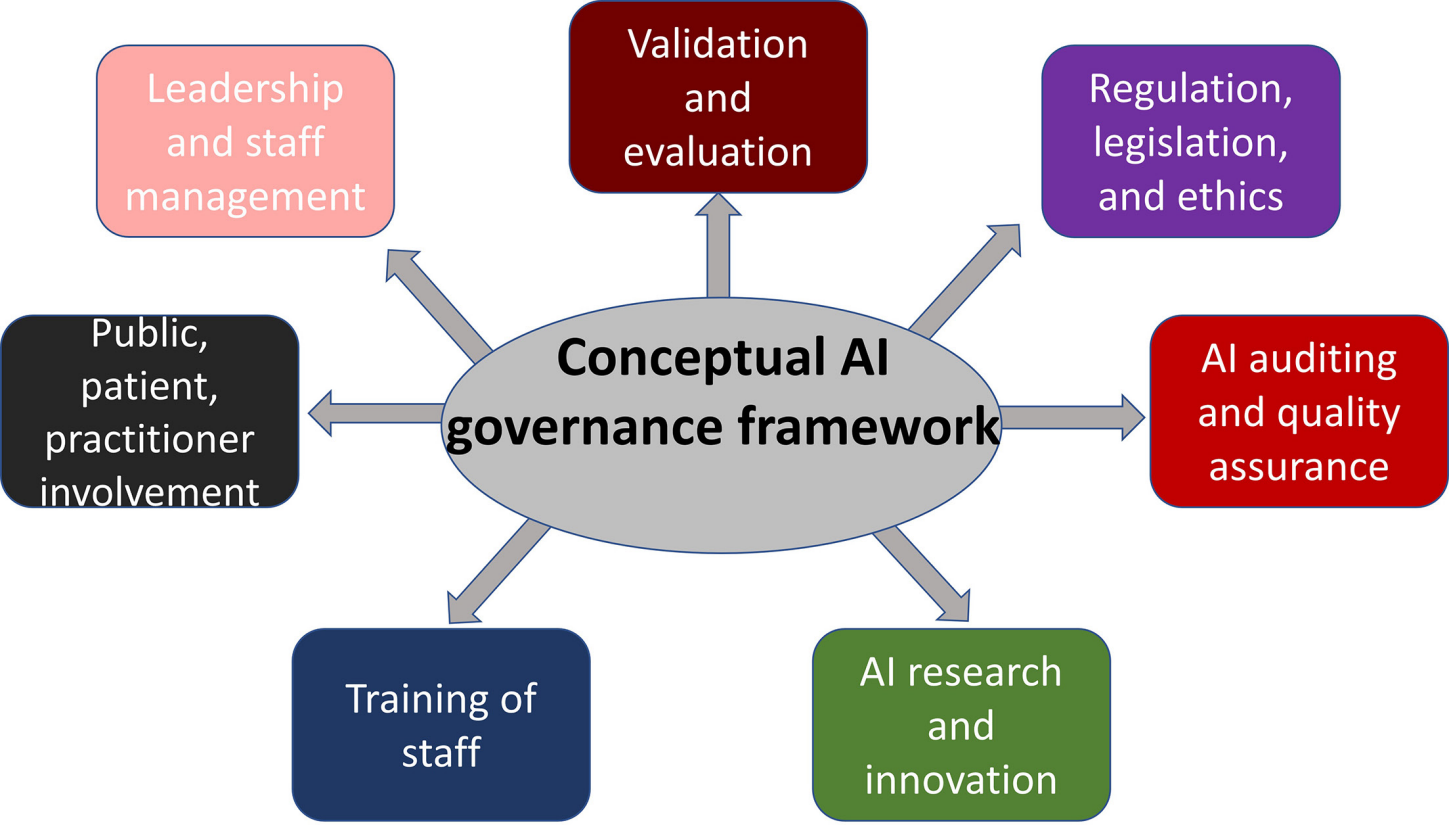
Suggested AI governance framework. AI, artificial intelligence.

The main findings were classified into concepts, then grouped into categories and further synthesised into themes in line with a content analysis approach.^32^ The results of this scoping review enabled the researchers to propose a conceptual AI governance framework based on the most widely discussed topics and principles of AI governance. The following figure (Figure 3) demonstrates the seven pillars of AI governance, as identified in the literature and synthesised here into themes.

**Figure 4.**
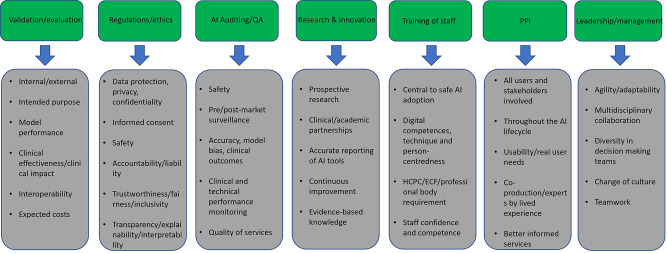
Important concepts under each pillar of the suggested AI governance framework.

The above AI governance framework includes some fundamental principles of AI governance, such as validation, evaluation, and auditing of AI systems, while also highlighting the importance of research and innovation, appropriate staff education, and effective leadership to ensure the safe and successful deployment of AI in clinical practice. Some of the most important categories, allowing finer detail of the suggested AI governance framework, as identified by this scoping review, are summarised in the following figure (Figure 4) and in the discussion.

## Discussion

### Validation and evaluation

Validation and evaluation were highlighted in the literature as vital to assess an AI model’s technical performance and clinical effectiveness before clinical deployment,^33,34^ ideally using multiple metrics and appropriate statistical methods^35^ to ensure that the model is aligned with its intended purpose.^36–38^ Evidence suggests the need for internal and external validation using unseen data. Validation has also been used in other contexts, *e.g.* to assess the generalisability/interoperability of the model.^39,40^

Standardised, rigorous validation protocols and curated databases have been developed to facilitate this process.^41^ Assessment of the clinical effectiveness of an AI model examines both its ability to generate the intended output effectively and to have a meaningful clinical impact.^34,42^ Hence, a 3-phase validation process has been proposed, consisting of validation against the test set of the data set (internal validation), validation against unseen data (external validation), and validation against diverse datasets from multiple centres.^35^

While this might sound optimal for diagnostic accuracy and clinical efficiency, in reality, data privacy and security when it comes to training and testing AI tools often make large, diverse, multisite data sets inaccessible to developers.^43^

AI model interoperability enables seamless data flow across different imaging centres, systems, or geographical areas.^42,44^ Interoperability in hardware, software, and data use can be achieved if large, representative training data sets are employed.^45^ A common interoperability software framework has been suggested in clinical imaging.^41^

Estimation of projected additional costs compared to standard practice^46^ and their impact on the use of available resources^39^ are also central to a comprehensive evaluation process.

### Regulations, legislation, and ethics

The papers reinforce the need for rigorous regulatory frameworks to safely use any AI-enabled medical devices; however, stringent standards may come at the cost of limiting innovation.^44^ Regulations must be applied to data protection, safety, and ethical use of AI models.^47^ However, regulatory frameworks have not yet been standardised in many countries^48^ due to technological advancements.^49^

In the EU, all medical devices must be classified according to their risk and conform to the required CE regulations.^39^ In the UK, all medical devices should be registered with the MHRA and undergo a UK Conformity Assessment (UKCA) from July 2024 due to the UK’s exit from the EU.^50^ CE-marked devices will be accepted in the UK until that date. Devices CE-marked under the Medical Devices Directive (before 2021) will have a further 3 years to gain a UKCA certification, and devices CE-marked under the Medical Devices Regulation will have a further 5 years. In addition, all medical devices should also comply with the General Data Protection Regulation and, in the UK, the NHS Digital Technologies Assessment Criteria framework.^20,42^ Moreover, all AI models should be classified according to their intended purpose, and this classification should be based on their potential risk to the system and service users.^46^

Data protection is paramount when using AI technologies; all organisations must safely use, store, and dispose of data while upholding privacy and confidentiality.^42,51–55^

Informed consent is another vital element of AI governance relating to the right of every patient/data owner to be informed about when their data will be used and for what purpose.^56^ Consent reinforces the right to human autonomy.^53,57^ Also, informed consent from data owners must be a dynamic process, and data owners should have the right to access their data and decide how it can be accessed by third parties.^51^

In addition, all organisations must take appropriate steps to ensure data safety against potential adversarial attacks since data collection and usage raises concerns regarding cybersecurity.^51^ Safety might also be compromised by the ‘algorithm update problem’ or ‘concept drift’, which can impact the algorithm’s performance over time as new data comes in.^58^ Algorithms could be locked to remain static, limiting usability; a solution to this challenge could be to assess the algorithms for any changes periodically.^44,58^ Ongoing data safety can be challenging and costly to maintain but necessary.

Serious concerns have been raised regarding potential patient harm from poor use or insufficient validation of AI models.^59^ Liability of health practitioners is associated mainly with medical malpractice and negligence, while developers’ liability would most likely fall under product design liability.^59^ Clinical practitioners may be liable for implementing inappropriate or non-validated AI tools in clinical practice or for failing to substantiate their recommendations.

All AI models must address the principle of fairness^36,39,51^ and avoidance of harm by minimising bias.^45^ For AI models to be fair, it is essential to ensure equity of benefits and costs and eliminate discrimination, stigmatisation, and unfair bias.^36^ Algorithmic biases are often due to non-diverse training data sets or testing only on specific population groups.^44,60^ AI models are prone to discrimination biases, which have been confirmed to be an important ethical issue.^53^

In addition, AI models should be transparent to be fair, inclusive, and easy to evaluate. Transparency requires AI models to be always available to interrogation.^47,61^ Also, transparent AI solutions will help build trust between patients and healthcare providers and between developers and clinical practitioners as end users.^52^ Another way to increase the transparency of AI models is to establish good traceability. This principle refers to the standardised documentation of all development processes, including data collection, labelling, devices used, and annotation tools.^44^

Furthermore, AI models must be explainable so that the patients and the trained staff can understand the reasoning behind their decision-making process.^39^ This could enhance trust between end users and AI technologies. Explainability is closely associated with transparency.^38,58^

In contrast, interpretability, often confused with explainability, refers to the ability of a model to make correct associations between cause and effect. Interpretability increases when AI models are explainable, although these models may exhibit reduced performance due to becoming more prone to external manipulation.^48^

### AI auditing and quality assurance

All organisations must develop ongoing procedures to test the AI model’s performance throughout its life cycle.^20^ Real-world performance monitoring, in the form of pre- and post-market surveillance, has been suggested to assess any deviation in the model’s performance over time.^40^ These procedures should focus on load tests, safety, bias testing^20^ and clinical and technical performance over time. Regular audits have been recommended to test the clinical safety of these models, particularly after model updates. Reporting of these audits should include accuracy, model biases, and clinical outcomes.^48^ All vendors should outline appropriate plans to assess their model’s performance drifts, automatically install any necessary updates and mitigate any risks from these updates.^39^

### AI research and innovation

Research is fundamental for improving clinical practice, patient outcomes, staff well-being and optimising workflows.^56^ However, there is still a lot to be done to ensure prospective studies are prioritised and that links between industry and academia are strengthened.^62^ With more academic–industry partnerships, it is vital to ensure the impartiality of researchers; researcher and clinician internships in AI startups will be central to supporting ethical, person-centred research.^62^

Prospective research studies are essential to assess and document the real added value of AI in healthcare.^46^ There is a need to evaluate AI tools after implementation, and there are already some checklists to assess research quality and risk of bias.^46^ The Standard Protocol Items: Recommendations for Interventional Trials-AI and Consolidated Standards of Reporting Trials-AI guidelines have been developed to increase the quality of conduct and reporting of AI-related clinical trials.^63^ In addition, the Checklist for AI in Medical Imaging guidelines facilitates medical imaging research reporting around AI research,^64^ while a quality score has been developed for radiomic studies. Finally, new reporting guidelines have been recommended to evaluate AI interventions moving from the algorithm development stage to support large clinical trials.^65^

### Training of staff

Training healthcare staff on AI principles has been hailed as central to AI adoption. The HCPC has recently advised that AI digital competencies are paramount for training radiographers to practise safely and care for patients. AI as a core competency is also embedded in the latest education and career framework and the recent AI guidance by the Society and College of Radiographers and other professional and regulatory bodies. This training should include knowledge about AI basic principles, validation and evaluation, clinical applications, governance and ethics, regulation and technology implementation, and the model’s limitations. The training should include principles of person-centred care and precision medicine.^48^ Appropriate staff training on AI technologies enables them to build confidence in effectively and safely using these AI tools^20^ for improved workflows, better patient outcomes and higher job satisfaction. In addition, it was found that appropriate training/education provided to healthcare professionals will also increase trustworthiness.^45^

### Public, patient, and practitioner involvement (PPI)

Many AI developers, unfortunately, seek user feedback retrospectively. Still, the cost of the afterthought can be huge both for the service-user and the organisation and could render AI adoption impractical. Prospective user, patient and public involvement should be included at all stages, from design to product roll-out and throughout its life cycle.^50^ Clinical usability is vital when deploying AI tools since user-friendly interfaces, and accessible, inclusive applications are central to effective AI adoption.^42,66^

Key stakeholders of an AI solution must be actively involved at all stages of the AI integration process.^39^ Staff involvement will ensure that the AI tool will meet their ergonomic, workflow and performance needs, as well as the needs of their patients. Also, participation in decisions affecting people’s lives is a core human right, requiring access to information and freedom of expression.^55^ Moreover, patients and the community must also be involved in AI product design and delivering of solutions to clinical problems. They are experts through lived experience and have unique insights into the challenges of workflows and usability of clinical tools.^39^ Public and patient representatives will be more effective if they are invited to act as research co-producers^48^ rather than as reviewers of research outcomes at later stages. Key stakeholders may include clinicians, patients, operational and administrative leaders, hospital administrators, and regulatory agencies.^33^ AI adoption relies on a diverse, highly engaged, well-trained AI ecosystem.

### Leadership and staff management

Effective leadership is vital for supporting any new venture in healthcare and beyond, which is true for any successful AI adoption initiative. A well-informed, agile senior leadership should identify and support AI champions for change of culture and knowledge transfer in key practice areas.^20^ Furthermore, it is essential to enable diverse, multidisciplinary teams to carry the work forward and not rely on only some professionals.^41^

### Importance of AI governance frameworks

This review underlines that AI governance is multiparametric, and all elements must be finely tuned to safely and effectively deploy AI models in clinical practice. The need for rigorous governance frameworks has been suggested in healthcare and other contexts, such as public administration,^67^ finance,^68^ and academia.^69^ AI governance frameworks will also play a fundamental role in mitigating the risks associated with using AI models while also allowing to maximise their benefits,^70^ enable safe implementation,^71^ and build trust between humans and AI.^72^ For the seamless adoption of AI, training is essential.^16^ Whilst radiologists have been leading on designing and delivering AI-related educational initiatives,^73,74^ radiographers have just started investing in AI education initiatives in medical imaging,^20,21,75^ as central to adoption.^14,15,17^

### Pre- and post-market considerations

The main challenges associated with adopting AI solutions can be classified into pre- and post-market considerations. Before procurement of an AI model, it is crucial to know its purpose and clearly define the clinical problem.^76^ This will ensure that this model fits the needs of the organisation and the end-users. In addition, another crucial pre-market consideration is ensuring that the AI model has been thoroughly validated and that appropriate checks have been performed onsite to assess the model’s suitability.^77^ Furthermore, end-users should ensure that the AI solution does not discriminate against vulnerable groups and that appropriate measures have been taken to mitigate algorithmic bias.^78^ Finally, all AI models should be assessed based on regulatory standards, and caution should be paid to the required regulatory aspects (*e.g.* CE marks) to be in place.^71^

Post-market assessment of the model’s clinical safety and effectiveness is paramount to ensure that the model will continue to perform as per the initial design and that no harm will impact end-users. Ongoing monitoring of AI tools is essential due to the dynamic nature of these environments; hence, evaluation of these systems should be thoroughly performed throughout their life cycle.^79^ This monitoring should be standardised.^80^ An important post-market consideration is the potential risk of algorithmic biases over time. Third parties should be assigned for clinical audits, as they have proved valuable in detecting the weaknesses of AI models.^81^ All healthcare professionals that use AI models should be able to timely recognise and escalate performance failures resulting from data shifts.^82^ AI governance principles should therefore be applied to AI models throughout their life cycle, from model development to decommissioning.^83^

### Financial considerations

Another essential part of AI solutions is the financial implications of clinically deploying these models. Healthcare systems are struggling after COVID-19, decimated by poor staffing and access to material resources.^84^ Before procuring AI models, a detailed cost/benefit analysis^85^ should aim to reduce costs and improve services to benefit the patients and healthcare staff.^86^ Different financial models in healthcare delivery mean different reimbursement models exist. So financial considerations will vary in other countries.

### Limitations

The nature of a scoping review means that the findings may be broad due to the general nature of the research question in scoping reviews.^87^ The framework is intentionally generic in nature, so it can be adjusted and contextualised to individual and local policies and circumstances. However, as evidence is still being developed around AI governance, this was the most appropriate methodology to gather the necessary evidence.

While Health Economics and Outcomes Research is central to AI adoption,^88^ it was not included in this review. This was partly because of the complexity of addressing this topic for different organisations. The little evidence starting to emerge, mainly from North America, shows that reimbursement analysis will be central to AI adoption.^89,90^ However, it remains to be seen how relevant this would be in the UK context.

Finally, since AI is a rapidly changing field, and the number of AI-related publications is growing exponentially, this review will inevitably miss some relevant published work nearer the time of acceptance and publication.

Given the paucity of a unified AI governance framework in medical imaging and radiotherapy in the UK, our results could be used as robust groundwork to develop locally comprehensive AI governance schemes. It is also opportune that a new British Standards Institution specification, currently in public consultation, will be released in the UK in the spring of 2023 to help clarify the finer details of AI adoption.^91^

## Conclusion

This scoping review identified the key elements of AI governance in the UK, focusing on medical imaging and radiotherapy. The proposed conceptual governance framework encompasses rigorous validation and evaluation procedures of AI tools, ongoing monitoring of these models' safety and clinical effectiveness, and compliance with the appropriate accreditation bodies and regulatory standards. The fundamental ethical principles associated with the safe use of AI tools should also be followed, and all AI models should be fair, transparent, trustworthy, and explainable. Staff should be confident about using AI tools in clinical practice. Appropriate staff training is necessary to build trust between AI and humans and ensure the acceptability of new technology. Effective leadership and staff management will further enhance the safe adoption of AI, while research also plays a fundamental role in driving sustainable innovation and growth.
