# Supplementary material for: Black box no more: a scoping review of AI governance frameworks to guide procurement and adoption of AI in medical imaging and radiotherapy in the UK
Source: Br J Radiol. 2023 Oct 3;96(1152):20221157. doi: 10.1259/bjr.20221157 (PMC10646619; doi:10.1259/bjr.20221157)
Supplement: Supplementary Material 1. [file bjr.20221157.suppl-01.docx]

**Keywords arranged in search strings:**

#1 Artificial Intelligence **OR** AI **OR** Machine Learning **OR** Deep Learning **OR** Digital Health

#2 Regulation **OR** regulat* **OR** governance **OR** framework **OR** rule* **OR** guidance **OR** guid* OR Accreditation OR standardization/standard*,OR Legislation, **OR** policy **OR** recommend*

#3 Data privacy **OR** ethic* **OR** integrity **OR** accountability

#4 Explainability **OR** transparency

#5 Transferability

#6 Interpretability

#7 Evaluation **OR** validation **OR** validat* **OR** ground truth

#8 Medical Imaging **OR** Radiology **OR** Radiography **OR** Radiotherapy **OR** Nuclear Medicine

**Search strategy:**

#1 **AND** #2 **AND** #8

#1 **AND** #3 **AND** #8

#1 **AND** #4 **AND** #8

#1 **AND** #5 **AND** #8

#1 **AND** #6 **AND** #8

#1 **AND** #7 **AND** #8
